# Multimodal Machine Learning in Image-Based and Clinical Biomedicine: Survey and Prospects

**DOI:** 10.1007/s11263-024-02032-8

**Published:** 2024-04-23

**Authors:** Elisa Warner, Joonsang Lee, William Hsu, Tanveer Syeda-Mahmood, Charles E. Kahn Jr., Olivier Gevaert, Arvind Rao

**Affiliations:** 1 Department of Computational Medicine and Bioinformatics, University of Michigan Ann Arbor, 100 Washtenaw Ave., Ann Arbor, MI 48109 USA; 2 Department of Medical and Imaging Informatics, University of California Los Angeles, 924 Westwood Blvd Ste 420, Los Angeles, CA 90024 USA; 3 Almaden Research Center, IBM, 650 Harry Rd., San Jose, CA 95120 USA; 4 Department of Radiology, University of Pennsylvania, 3400 Spruce St., Philadelphia, PA 19104 USA; 5 Center for Biomedical Informatics Research, Stanford, 1265 Welch Road, Stanford, CA 94305 USA

**Keywords:** Machine learning, Multimodal, Representation, Fusion, Translation, Alignment, Co-learning, Artificial intelligence, Data integration

## Abstract

Machine learning (ML) applications in medical artificial intelligence (AI) systems have shifted from traditional and statistical methods to increasing application of deep learning models. This survey navigates the current landscape of multimodal ML, focusing on its profound impact on medical image analysis and clinical decision support systems. Emphasizing challenges and innovations in addressing multimodal representation, fusion, translation, alignment, and co-learning, the paper explores the transformative potential of multimodal models for clinical predictions. It also highlights the need for principled assessments and practical implementation of such models, bringing attention to the dynamics between decision support systems and healthcare providers and personnel. Despite advancements, challenges such as data biases and the scarcity of “big data” in many biomedical domains persist. We conclude with a discussion on principled innovation and collaborative efforts to further the mission of seamless integration of multimodal ML models into biomedical practice.

## Introduction

Machine learning (ML), the process of leveraging algorithms and optimization to infer strategies for solving learning tasks, has enabled some of the greatest developments in artificial intelligence (AI) in the last decade, enabling the automated segmentation or class identification of images, the ability to answer nearly any text-based question, and the ability to generate images never seen before. In biomedical research, many of these ML models are quickly being applied to medical images and decision support systems in conjunction with a significant shift from traditional and statistical methods to increasing application of deep learning models. At the same time, the importance of both plentiful and well-curated data has become better understood, coinciding as of the time of writing this article with the incredible premise of OpenAI’s ChatGPT and GPT-4 engines as well as other generative AI models which are trained on a vast, well-curated, and diverse array of content from across the internet (OpenAI, 2023).

As more data has become available, a wider selection of datasets containing more than one modality has also enabled growth in the multimodal research sphere. Multimodal data is intrinsic to biomedical research and clinical care. While data belonging to a single modality can be conceptualized as a way in which something is perceived or captured in the world into an abstract digitized representation such as a waveform or image, multimodal data aggregates multiple modalities and thus consists of several intrinsically different representation spaces (and potentially even different data geometries). Computed tomography (CT) and positron emission tomography (PET) are specific examples of single imaging modalities, while magnetic resonance imaging (MRI) is an example itself of multimodal data, as its component sequences T1-weighted, T2-weighted, and fluid-attenuated inversion recovery (FLAIR) can each be considered their own unique modalities, since each of the MR sequences measure some different biophysical or biological property. Laboratory blood tests, patient demographics, electrocardiogram (ECG) and genetic expression values are also common modalities in clinical decision models. This work discusses unique ways that differences between modalities have been addressed and mitigated to improve accuracy of AI models in similar ways to which a human would naturally be able to re-calibrate to these differences.

There is conceptual value to building multimodal models. Outside of the biomedical sphere, many have already witnessed the sheer power of multimodal AI in text-to-image generators such as DALL$\cdot$E 2, DALL$\cdot$E 3 or Midjourney (Ramesh et al., 2022; Betker et al., 2023; Oppenlaender, 2022), some of whose artful creations have won competitions competing against humans (Metz, 2022). In the biomedical sphere, multimodal models provide potentially more robust and generalizable AI predictions as well as a more holistic approach to diagnosis or prognosis of patients, akin to a more human-like approach to medicine. While a plethora of biomedical AI publications based on unimodal data exist, fewer multimodal models exist due to cost and availability constraints of obtaining multimodal data. However, since patient imaging and lab measurements are decreasing in cost and increasing in availability, the case for building multimodal biomedical AI is becoming increasingly compelling.

**Fig. 1:**
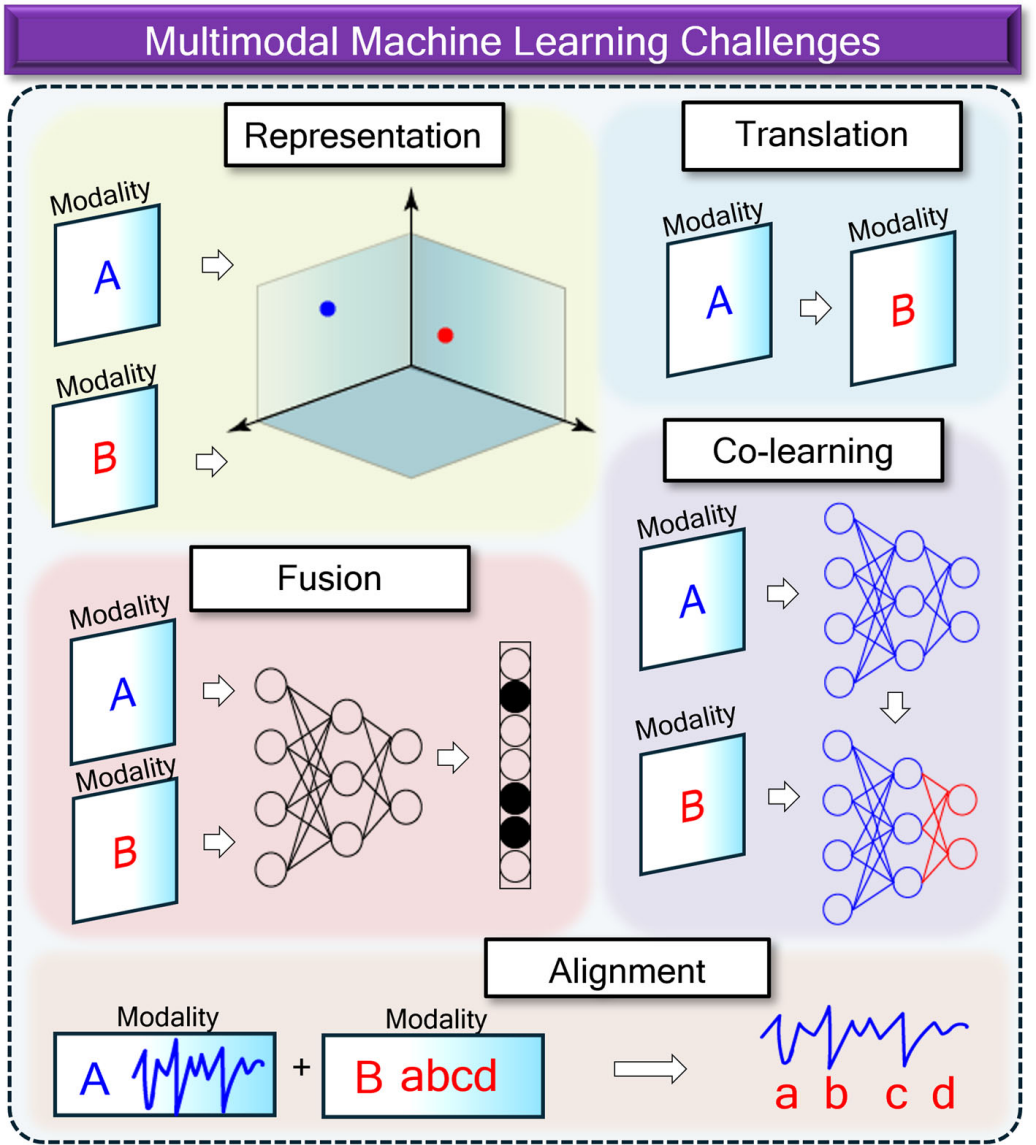
Challenges in multimodal learning: (1) representation, which concerns how multiple modalities will be geometrically represented and how to represent intrinsic relationships between them; (2) fusion, the challenge of combining multiple modalities into a predictive model; (3) translation, involving the mapping of one modality to another; (4) alignment, which attempts to align two separate modalities spatially or temporally; and (5) co-learning, which involves using one modality to assist the learning of another modality

With the emergence of readily-available multimodal data comes new challenges and responsibilities for those who use them. The survey and taxonomy from Baltrusaitis et al. (2019) presents an organized description of these new challenges, which can be summarized in Fig. 1: (1) representation, (2) fusion, (3) alignment, (4) translation, (5) co-learning. *Representation* often condenses a single modality such as audio or an image to a machine-readable data structure such as a vector, matrix, tensor object, or other geometric form, and is concerned with ways to combine more than one modality into the same representation space. Good multimodal representations are constructed in ways in which relationships and context can be preserved between modalities. Multimodal *fusion* relates to the challenge of how to properly combine multimodal data into a predictive model. In multimodal *alignment*, models attempt to automatically align one modality to another. In a simple case, models could be constructed to align PPG signals taken at a 60Hz sampling frequency with a 240Hz ECG signal. In a more challenging case, video of colonoscopy could be aligned to an image representing the camera’s location in the colon. Multimodal *translation* consists of mapping one modality to another. For example, several popular natural language processing (NLP) models attempt to map an image to a description of the image, switching from the imaging domain to a text domain. In translational medicine, image-to-image translation tends to be the most common method, whereby one easily-obtained imaging domain such as CT is converted to a harder-to-obtain domain such as T1-weighted MRI. Lastly, multimodal *co-learning* involves the practice of transferring knowledge learned from one modality to a model or data from a different modality.

In this paper, we use the taxonomical framework from Baltrusaitis et al. (2019) to survey current methods which address each of the five challenges of multimodal learning with a novel focus on addressing these challenges in medical image-based clinical decision support. The aim of this work is to introduce both current and new approaches for addressing each multimodal challenge. We conclude with a discussion on the future of AI in biomedicine and what steps we anticipate could further progress in the field.

## Multimodal Learning in Medical Applications

**Fig. 2:**
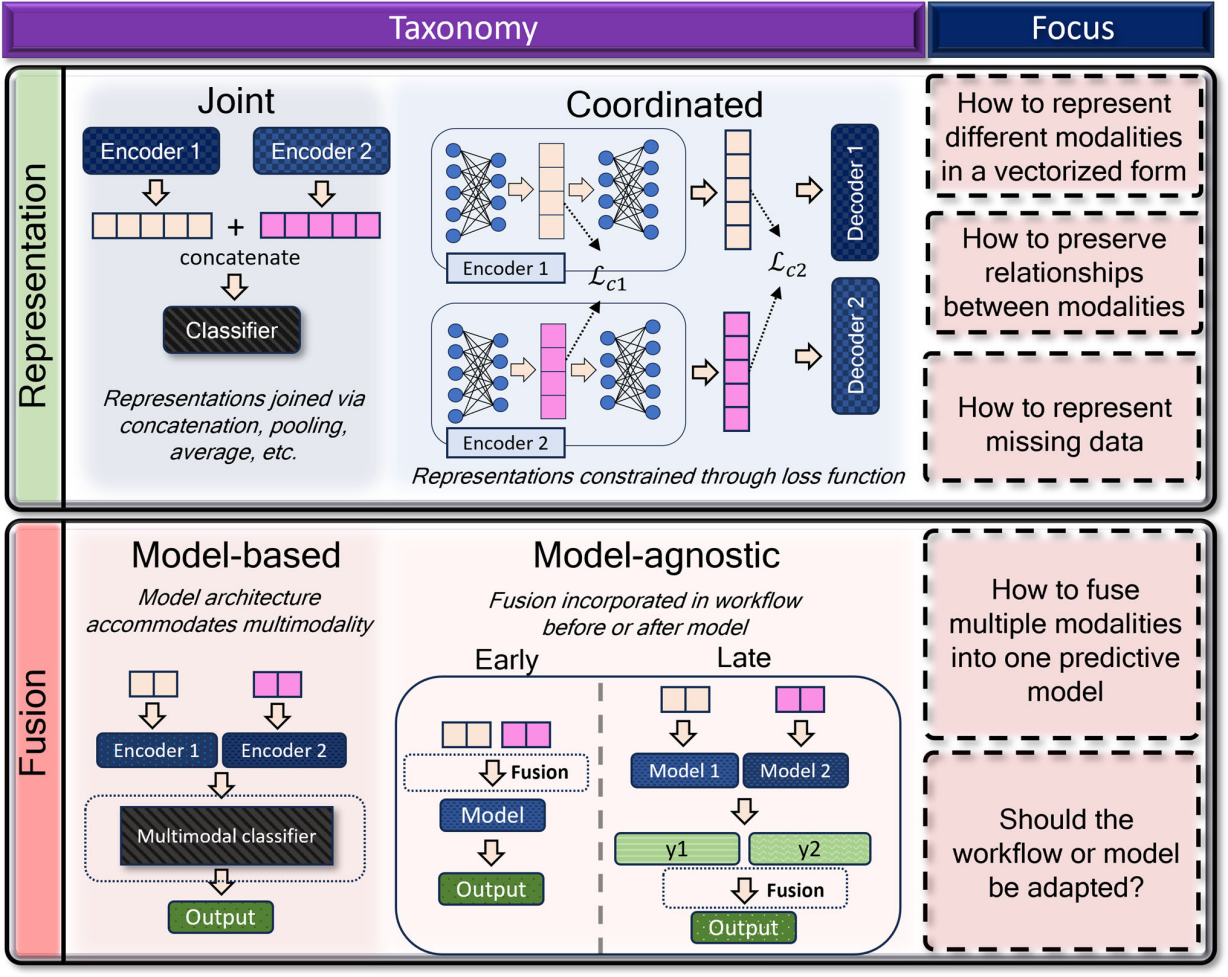
A graphical representation of the taxonomical sublevels of multimodal representation and fusion, and the focus of each challenge. Multimodal representation can be categorized into whether the representations are joined into a single vector (*joint*) or separately constructed to be influenced by each other (*coordinated*). Multimodal fusion can be distinguished by whether a model is uniquely constructed to fuse the modalities (*model-based*), or whether fusion occurs before or after the model step (*model-agnostic*)

**Fig. 3:**
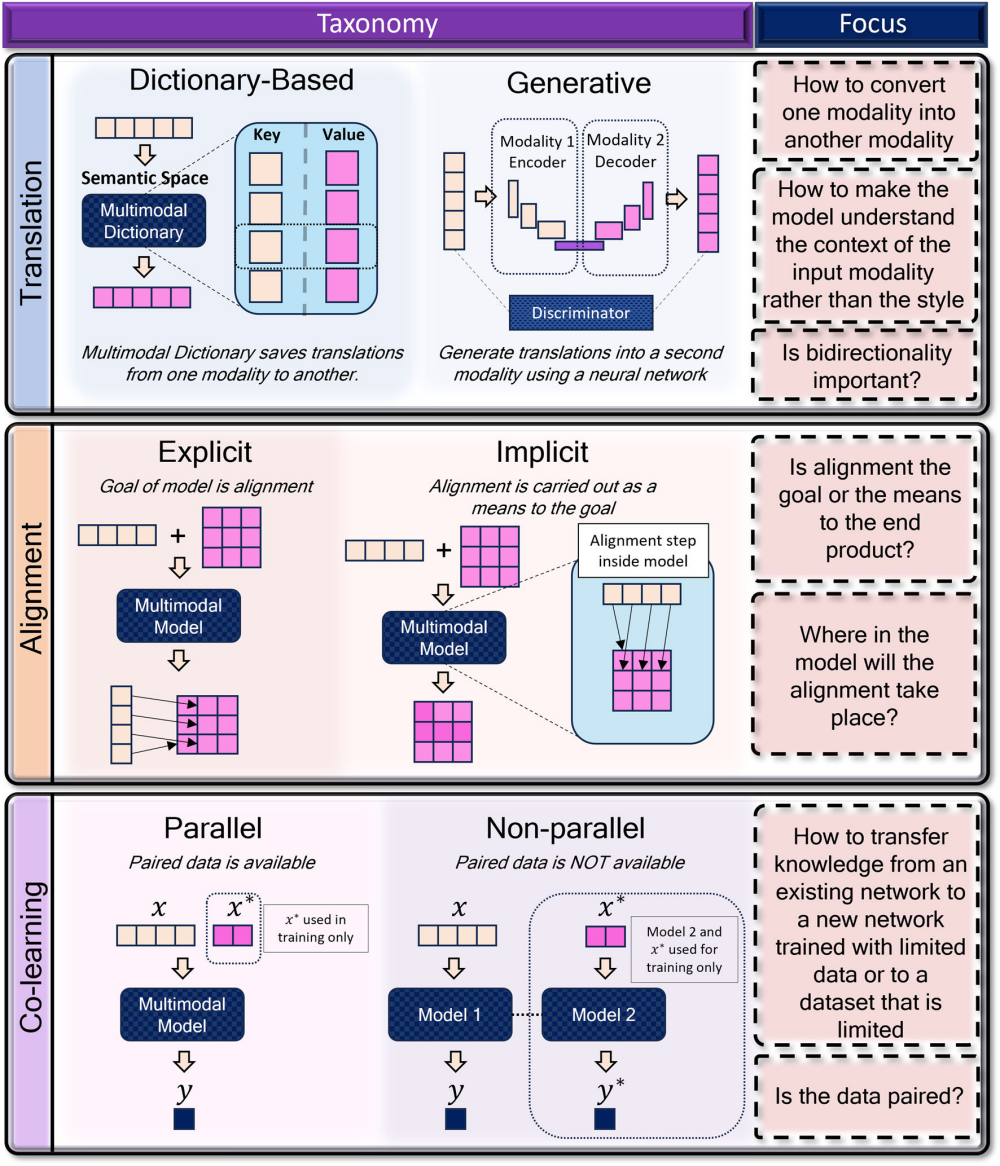
A graphical representation of the taxonomical sublevels of multimodal translation, alignment and co-learning, and the focus of each challenge. In *translation*, models are distinguished based on whether they require use of a dictionary to save associations between modalities (*dictionary-based*), or if the associations are learned in a multimodal network (*generative*). In *alignment*, distinction is made depending on the *purpose* of the alignment, whether as the goal (*explicit*) or as an intermediate step towards the goal output (*implicit*). In *co-learning*, a distinction is made between the use of *parallel* (paired) multimodal data, or *non-parallel* (unpaired) multimodal data. In co-learning models, one of the modalities is only used in training but does not appear in testing

In the following section, we reintroduce the five common challenges in multimodal ML addressed in Sect. 1 and discuss modern approaches to each challenge as applied to image-based biomedicine. The taxonomical subcategories of Representation and Fusion are summarized in Fig. 2, while those for Translation, Alignment and Co-learning are summarized in Fig. 3. A table of relevant works by the challenge addressed and data types used are given in Table 1.

### Representation

Representation in machine learning typically entails the challenge of transferring contextual knowledge of a complex entity such as an image or sound to a mathematically-interpretable or machine-readable format such as a vector or a matrix. Prior to the rise of deep learning, features were engineered in images using techniques such as the aforementioned Scale-Invariant Feature Transform (SIFT) or through methods such as edge detection. Features in audio or other waveform signals such as ECG could be extracted utilizing wavelets or Fourier transform to isolate latent properties of signals and then quantitative values could be derived from morphological patterns in the extracted signal. Multimodal representation challenges venture a step further, consisting of the ability to translate similarities and differences from one modality’s representation to another modality’s representation. For example, when representing both medical text and CT images, if the vector representations for “skull” and “brain” in medical *text* are closer than those for “skull” and “pancreas”, then in a good CT representation, such relationships between vector representations of these structures in the *image* should remain preserved. The derivation of “good” representations in multimodal settings have been outlined in Bengio et al. (2013) and extended by Srivastava and Salakhutdinov (2014).

It is crucial to acknowledge that representation becomes notably challenging when dealing with more abstract concepts. In a unimodal context, consider the task of crafting representations from an image. Beyond pixel intensities, these representations must encapsulate contextual and semantically-proximate information from the image. A simplistic model may fail to encode context adequately, discerning insufficient distinctions between a foreground and background to represent nuanced visual-semantic concepts. Achieving such subtleties in representations, particularly in abstract contexts, poses increased challenges compared to quantifying similarities and differences in less-nuanced data such as cell counts or gene expression.

Prior to delving into multimodal representations, it is instructive to elucidate strategies for crafting proficient unimodal representations, as multimodal approaches often involve combining or adapting multiple unimodal methods. For images, *pretrained networks* are a common approach for transforming images into good vector representations. Another approach is use of autoencoders, which condense image representations into lower-dimensional context vectors that can be decoded to reconstruct the original image. *Multimodal autoencoders* have been applied to MRI modalities in Hamghalam et al. (2021) and in this example were also utilized to impute representations for missing modalities.

**Table 1:** Literature relating to the five challenges of multimodal machine learning by the datatype analyzed

	Datatype				
Challenge	MRI	CT	PET	EHR	
Representation	Hamghalam et al. (2021) and Zhang et al. (2022)	Daza et al. (2020) and Zhou et al. (2023)		Daza et al. (2020), Sonsbeek and Worring (2020), Zhang et al. (2023) and Wang et al. (2023)	
Fusion	Azcona et al. (2020), Neubauer et al. (2020), Zhou et al. (2020), Wang et al. (25021), Zhang et al. (2022), Rudie et al. (2022), Liu et al. (2023), Zhang et al. (2023) and Zhou et al. (2023)	Daza et al. (2020), Neubauer et al. (2020), Yang et al. (2020), Bhalodia et al. (2021) and Carbonell et al. (2023)	Neubauer et al. (2020)	Daza et al. (2020), Yang et al. (2020), Sonsbeek and Worring (2020), Zhou et al. (2020), Vivar et al. (2020), Li et al. (2021), Bhalodia et al. (2021), Cui et al. (2022) and Khosravi et al. (2022)	
Translation	Jiang and Veeraraghavan (2020), Hu et al. (2020), Guo et al. (2020), Shin et al. (2020) and Takagi and Nishimoto (2023)	Jiang and Veeraraghavan (2020) and Zhu et al. (2020)	Hu et al. (2020) and Shin et al. (2020)		
Alignment	Wang et al. (25021) and Zhang et al. (2022)	Yang et al. (2020), Leroy et al. (2023), Zhou et al. (2023) and Li et al. (2023)		Yang et al. (2020) and Li et al. (2023)	
Co-learning	Varsavsky et al. (2020), Yang et al. (2020), Hu et al. (2020), Bui et al. (2020), Hu et al. (2021), Pei et al. (2023), Jafari et al. (2022), Xue et al. (2020) and Dong et al. (2022)	Xue et al. (2020), Hu et al. (2021), Pei et al. (2023), Jafari et al. (2022) and Dong et al. (2022)		Xing et al. (2022)	

	Datatype				
Challenge	Hist	Ultrasound	Genomic	X-ray	Fundus
Representation				Sonsbeek and Worring (2020)	Zhou et al. (2023)
Fusion	Chen et al. (2020), Li et al. (2021) and Cui et al. (2022)	Habib et al. (2020)	Chen et al. (2020) and Cui et al. (2022)	Sonsbeek and Worring (2020), Habib et al. (2020), Cui et al. (2022), Carbonell et al. (2023), Khosravi et al. (2022)	Zhou et al. (2023)
Translation					
Alignment	Leroy et al. (2023)				Zhou et al. (2023)
Co-learning		Xiong et al. (2020)	Chauhan et al. (2020)		

Another approach for multimodal representation could be through the use of *disentanglement networks*, which can separate latent properties of an image into separate vectors. In such cases, an image is given as input and the autoencoder is often split in such a way that two vectors are produced as intermediate pathways, where joining the intermediate vectors should result in the original input. Each intermediate pathway is often constrained by a separate loss function term to encourage separation of each pathway into the desired latent characteristics. In this way, one input image can be represented by two separate vectors, each representing a disjointed characteristic of the image. This disentanglement method has been applied in Jiang and Veeraraghavan (2020) to separate context in CT and MRI from their style so that one modality can be converted in to the other. It was also applied for a single modality in Bône et al. (2020) to separate “shape” and “appearance” representations of an input, which could potentially be applied to different imaging modalities to extract only similar shapes from each.

When two or more vectorized modalities are combined into a model, they are typically combined in one of two ways: (1) joint, or (2) coordinated representations. A *joint representation* is characterized by aggregation of the vectors at some point in the process, whereby vector representations from two separate modalities are joined together into a single vector form through methods such as aggregation, concatenation or summation. Joint representation is both a common and effective strategy for representation; however, a joint strategy such as concatenation is often constricted to serving in situations where both modalities are available at train- and test-time (one exception using Boltzmann Machines can be found in Srivastava and Salakhutdinov (2014)). If a modality has the potential to be missing, a joint strategy such as aggregation via weighted means could be a better option (Li et al., 2021; Chen et al., 2020; Zhou et al., 2023; Cui et al., 2022). Using mathematical notation from Baltrusaitis et al. (2019), we can denote joint representations $x_m$ as the following:

1 $$\begin{aligned} x_m=f(x_1,...,x_n) \end{aligned}$$

This denotes that feature vectors $x_i, i =1...n$ are combined in some way through a function *f* to create a new representation space $x_m$. By the contrary, *coordinated representations* are represented as the following:

2 $$\begin{aligned} f(x_1)\sim g(x_2), \end{aligned}$$

whereby a function designed to create representations for one modality may be constrained (represented by $\sim$) by a similar function from another modality, with the assumption that relationships between data points in the first modality should be relatively well-preserved in the second modality.

Joint representations tend to be the most common approach to representing two or more modalities together in a model because it is perhaps the most straightforward approach. For example, joining vectorized multimodal data together through concatenation before entering a model tends to be one of the most direct approaches to joint representation. Sonsbeek and Worring (2020), for example, chest x-rays are combined with text data from electronic health records in a vectorized form using a pretrained model first. Then, the vectors from each modality are sent individually through two attention-based blocks, then concatenated into a joint feature space to predict a possible cardiovascular disease and generate a free-text “impression” of the condition. Other joint representation models follow simpler methods, simply extracting baseline features from a pretrained model and concatenating them Daza et al. (2020); Yang et al. (2020).

Although coordinated representations have traditionally tended to be more challenging to implement, the convenience of neural network architectural and loss adjustments have resulted in increased traction in publications embodying coordinated representations (Xing et al., 2022; Wang et al., 2023; Chauhan et al., 2020; Radford et al., 2021; Zhang et al., 2022; Bhalodia et al., 2021). One of the most notable of these in recent AI approaches is OpenAI’s Contrastive Language-Image Pre-Training (CLIP) model, which forms representations for OpenAI’s DALL$\cdot$E 2 (Radford et al., 2021; Ramesh et al., 2022) and uses a contrastive-learning approach to shape both image embeddings of entire images to match text embeddings of entire captions describing those images. The representations learned from CLIP were demonstrated to not only perform well in zero-shot image-to-text or text-to-image models, but also to produce representations that could outpace baseline supervised learning methods. In a biomedical context, similar models abound, including ConVIRT, a predecessor and forerunner for CLIP (Zhang et al., 2022), and related works (Bhalodia et al., 2021).

Coordinated approaches are especially useful in co-learning. Chauhan et al. (2020), which employs a subset of co-learning called privileged information, the geometric forms of each modality are not joined into a single vector representation. Instead, network weights are encouraged to produce similar output vectors for each modality and ultimately the same classifications. This constraint warps the space of chest x-ray representations closer to the space of text representations, with the assumption that this coordinated strategy provides chest x-ray representations more useful information because of the text modality. For more on privileged information, see the Sect. 2.5 below.

In the biomedical sphere, where models are built to prioritize biologically- or clinically-relevant outcomes, quality of representations may often be overlooked or overshadowed by emphasis on optimization of prediction accuracy. However, there is conceptual value in building good multimodal representations. If models are constructed to ensure that similar concepts in different modalities also demonstrate cross-modal similarity, then there is greater confidence that an accurate model is understanding cross-modal relationships. While building good cross-modal representations for indexing images on the Internet like in the CLIP model is a digestible challenge, building similar cross-modal representations for medical data presents a far more formidable challenge due to data paucity. OpenAI’s proprietary WebTextImage dataset, used for CLIP, contains 400 million examples, a sample size that is as of yet unheard of for any kind of biomedical imaging data. Until such a dataset is released, bioinformaticians must often rely on the ability to leverage pretrained models and transfer learning strategies for optimal results amidst low resources to leverage big data for good representations on smaller data.

### Fusion

Next, we discuss challenges in multimodal fusion. This topic is a natural segue from the discussion of representation because many multimodal representations are subsequently fed into a discriminatory model. Multimodal fusion entails the utilization of methods to combine representations from more than one modality into a classification, regression, or segmentation model. According to Baltrusaitis et al. (2019), fusion models can be classified into two subcategories: model-agnostic and model-based approaches. The term *“model-agnostic”* refers to methods for multimodal fusion occurring either before or after the model execution and typically does not involve altering the prediction model itself. Model-agnostic approaches can further be delineated by the stage at which the fusion of modalities occurs, either early in the model (prior to output generation) or late in the model (such as ensemble models, where outputs from multiple models are combined). Additionally, hybrid models, incorporating a blend of both early and late fusion, have been proposed (Carbonell et al., 2023). In contrast, a *model-based* approach entails special adjustments to the predictive model to ensure it handles each modality uniquely.

While model-agnostic methods remain pertinent as useful strategies for multimodal fusion, the overwhelming popularity of neural networks has led to a predominant shift towards model-based methods in recent years. These model-based methods involve innovative loss functions and architectures designed to handle each modality differently. One common model-based fusion strategy is multimodal *multiple instance learning (MIL)*, where multiple context vectors for each modality are generated and subsequently aggregated into a single representation leading to the output classification. The method for aggregation varies across studies, with attention-based approaches, emphasizing specific characteristics of each modality, being a common choice (Li et al., 2021; Chen et al., 2020; Zhou et al., 2023; Cui et al., 2022).

The construction of a good model architecture is crucial; however, challenges associated with fusion are often highly contextual, and thus it is important to understand what kinds of data are being utilized in recent models and what problems they try to solve. Most multimodal models understandably incorporate MRI modalities, given that MR images are a natural multimodal medium. Consequently, studies incorporating MRI such as Azcona et al. (2020), which aims to classify Alzheimer’s Disease severity, and Zhou et al. (2020), predicting overall survival in brain tumor patients, exemplify the type of research often prevalent in multimodal image-based clinical application publications. Brain-based ML studies are also popular because of the wide availability of brain images and a strong interest in applying ML models in clinical neuroradiology. However, recent models encompass a myriad of other clinical scenarios predicting lung cancer presence (Daza et al., 2020), segmenting soft tissue sarcomas (Neubauer et al., 2020), classifying breast lesions (Habib et al., 2020), and predicting therapy response (Yang et al., 2020), among others, by amalgamating and cross-referencing modalities such as CT images (Daza et al., 2020; Neubauer et al., 2020), blood tests (Yang et al., 2020), electronic health record (EHR) data (Yang et al., 2020; Sonsbeek and Worring, 2020; Daza et al., 2020), mammography images (Habib et al., 2020), and ultrasound (Habib et al., 2020).

Multimodal fusion models are emerging as the gold standard for clinical-assisted interventions due to the recognition that diagnosis and prognosis in real-world clinical settings are often multimodal problems. However, these models are not without limitations. For one, standardization across equipment manufacturers or measurement protocols can affect model performance dramatically, and this issue becomes more pronounced as more modalities are incorporated into a model. Second, while fusion models attempt to mimic real-world clinical practice, they face practical challenges that can limit their utility. For instance, physicians may face various roadblocks to obtaining all model input variables due to a lack of permission from insurance companies to perform all needed tests or time constraints. These issues underscore challenges associated with missing modalities, and several studies have attempted to address this concern (Carbonell et al., 2023; Zhang et al., 2022; Cui et al., 2022; Wang et al., 2023; Liu et al., 2023). However, incorporating mechanisms to account for missing modalities in a model is not yet a common practice for most multimodal biomedical models.

Lastly, many models are not configured to make predictions that adapt with additional variables. Most models necessitate all variables to be present at the time of operation, meaning that, even if all tests are conducted, the model can only make a decision once all test results have been obtained. In conclusion, in the dynamic and fast-paced environment of hospitals and other care centers, even accurate models may not be suitable for practical use, unless also coupled with mechanisms to handle missing data.

### Translation

In multimodal translation, a model is devised to operate as a mapping entity facilitating the transformation from one modality to another. This involves the conversion of input contextual data, such as CT scans, into an alternative contextual data format, such as MRI scans. Before the rise of modern *generative* methods leveraging multimodal generative adversarial networks (GANs) or diffusion models to generate one modality from another, translation via *dictionary-based* methods was common, which typically involved a bimodal dictionary whereby a single entry would contain a key belonging to one modality and a corresponding value belonging to the other modality. Dictionary-based translation was uncommon in biomedical research but popular in NLP fields as a way to convert images into text and vice versa (Liao et al., 2022; Reed et al., 2016). The current ascendancy of generative models and the availability of associated coding packages have since catalyzed the growth of innovative translational studies applying generative approaches.

Presently, generative models encompass a broad spectrum of potential applications both within and beyond the biomedical domain. Outside the medical sphere, generative models find utility in NLP settings, particularly in text-to-image models like DALL$\cdot$E 2 and Midjourney (Liao et al., 2022; Ramesh et al., 2022; Oppenlaender, 2022). Additionally, they are employed in style transfer and other aesthetic computer vision techniques (Huang et al., 2021; Cao et al., 2018; Zhu et al., 2017; Liu et al., 2018; Palsson et al., 2018; Zhang and Wang, 2020). Within the biomedical realm, generative models have proven efficacious in creating virtual stains for unstained histopathological tissues which would typically undergo hemotoxylin/eosin staining (Lu et al., 2021). Furthermore, these models serve as prominent tools for sample generation (Tseng et al., 2017; Piacentino et al., 2021; Choi et al., 2017), particularly in scenarios with limited sample sizes (Chen et al., 2021). Despite the potential diversity of multimodal translation involving any two modalities, predominant translational efforts in the biomedical realm currently revolve around mapping one imaging modality to another, a paradigm recognized as image-to-image translation.

In the contemporary landscape, the integration of simplistic generative models into a clinical context are declining in visibility, while methods employing specialized architectures tailored to the involved modalities are acknowledged for advancing the state-of-the-art in translational work. Within this context, two notable generative translation paradigms for biomedicine are explored: (1) medical image generation models, and (2) segmentation mask models. In the former, many studies attempt to form models that are bidirectional, whereby the intended output can be placed back as input and return an image similar to the original input image. Bui et al. (2020), this is resolved by generating deformation fields that map changes in the T1-weighted sequence modality of MRI to the T2-weighted sequence modality. Hu et al. (2020), separate forward and backward training processes are defined whereby an encoder representing PET images is utilized to understand the underlying distribution of that modality, allowing for more realistic synthetic generated images from MRI. In one unidirectional example, Shin et al. (2020) modifies a pix2pix conditional GAN network to allow Alzheimer’s disease classification to influence synthetic PET image generation. In another interesting example, Takagi and Nishimoto (2023) use functional MRI (fMRI) scans and diffusion models to attempt to recreate images of what their subjects had seen. Similarly, diffusion models and magnetoencephalography (MEG) are utilized by Meta for real-time prediction from brain activity of what patients had seen visually (Benchetrit et al., 2023).

In the second potential application, image segmentation models in multimodal image-to-image translation must handle additional challenges, creating both a way to generate the output modality as well as a way to segment it. Jiang and Veeraraghavan (2020), a generative model converts CT to MRI segmentation. In a reverse problem to image segmentation, Guo et al. (2020) attempts to synthesize multimodal MRI examples of lesions with only a binary lesion mask and a multimodal MRI Atlas. In this study, six CNN-based discriminators are utilized to ensure the authentic appearance of background, brain, and lesion, respectively, in synthesized images.

Multimodal translation still remains an exciting but formidable challenge. In NLP and beyond, there have been remarkable successes observed in new image generation within text-to-image models beyond the biomedical sphere. However, the adoption of translation models in biomedical work is evolving at a more measured pace, with applications extending beyond demonstrative feasibility to practical utility remaining limited. Arguments in favor of biomedical translation models are predominantly centered around sample generation for datasets with limited sizes, as the generated medical images must adhere to stringent accuracy requirements. Similar to other challenges in multimodal research, translation models would greatly benefit from training on more expansive and diverse datasets. However, with the increasing digitization of medical records and a refined understanding of de-identification protocols and data sharing rights, the evolution of this field holds considerable promise.

### Alignment

Multimodal alignment involves aligning two related modalities, often in either a spatial or temporal way. Multimodal alignment can be conducted either *explicitly* as a direct end goal, or *implicitly*, as a means to the end goal, which could be translation or classification of an input. One example of *explicit alignment* in a biomedical context is image registration. Leroy et al. (2023) highlights one approach to multimodal image registration, where histopathology slides are aligned to their (*x*, *y*, *z*) coordinates in a three-dimensional CT volume. Another is in Chen et al. (2023), where surgical video was aligned to a text description of what is happening in the video. On the other hand, an example of multimodal *implicit alignment* could be the temporal alignment of multiple clinical tests to understand a patients progress over time. Such an analysis was conducted in Yang et al. (2020), where the authors built a customized multi-layer perceptron (MLP) called SimTA to predict response to therapy intervention at a future time step based on results from previous tests and interventions.

Literature surrounding alignment has increased since the rise of attention-based models in 2016. The concept of “attention,” which relates to aligning representations in a way that is contextually relevant, is a unimodal alignment paradigm with origins in machine translation and NLP (Bahdanau et al., 2015). An example use of attention in NLP could be models which try to learn, based on order and word choice of an input sentence, where the subject of the sentence is so that the response can address the input topic. In imaging, attention can be used to highlight important parts of an image that are most likely to contribute to a class prediction. Vaswani et al. (2017), introduced a more sophisticated attention network, named transformers, an encoder-decoder-style architecture based on repeated projection heads where attention learning takes place. Transformers and attention were originally applied to natural language (Vaswani et al., 2017; Bahdanau et al., 2015; Devlin et al., 2019) but have since been applied to images (Parmar et al., 2018; Dosovitskiy et al., 2021), including histopathology slides (Lu et al., 2021; Chen et al., 2020) and protein prediction (Tunyasuvunakool et al., 2021). *Multimodal transformers* were introduced in 2019, also developed for the natural language community (Tsai et al., 2019). While these multimodal transformers do not contain the same encoder-decoder structure of a traditional transformer architecture, they are hallmarked by crossmodal attention heads, where one modality’s sequences intermingle with another modality’s sequences.

Although typical transformers themselves are not multimodal, they often constitute in multimodal models. The SimTA network mentioned above borrowed the positional encoding property of transformers to align multimodal inputs in time to predict therapy response (Yang et al., 2020). Many models taking advantage of visual transformers (ViT) have also utilized pretrained transformers trained on images for multimodal fusion models. In both the TransBTS (Wang et al., 25021) and mmFormer models (Zhang et al., 2022), a transformer is utilized on a vector composed of an amalgamation of information from multiple modalities of MRI, which may imply that the transformer attention heads here are aligning information from multiple modalities represented via aggregate latent vectors. The ultimate function of transformers is a form of implicit alignment, and it can be assumed here that this alignment is multimodal.

**Fig. 4:**
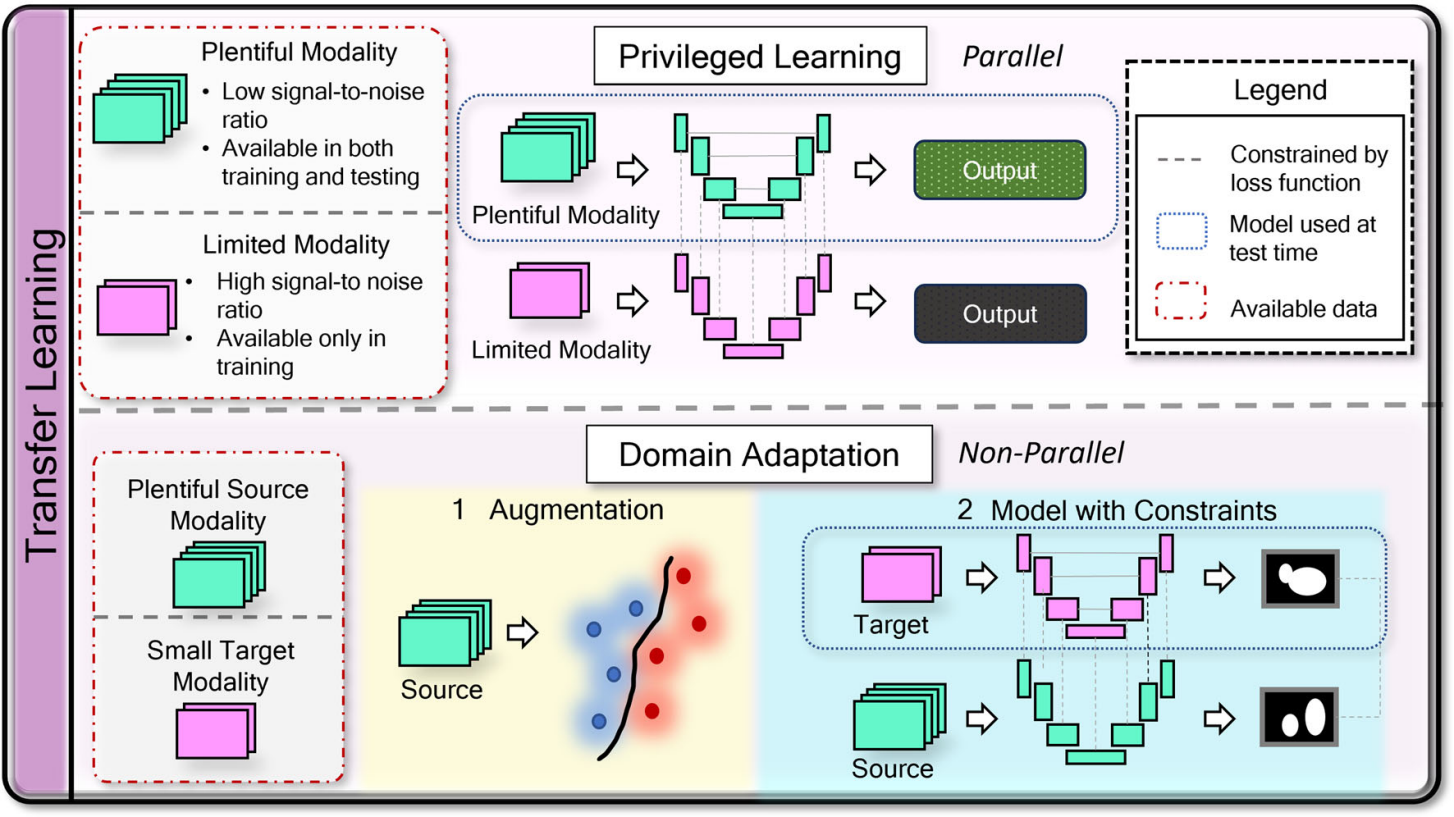
Two types of transfer learning described in this work are privileged learning (top) and domain adaptation (bottom). In privileged learning, a plentiful set consisting of data which is normally of low cost but also low signal-to-noise ratio is available in both training and testing, while a limited gold-standard quality set is used for training only. In this example, the plentiful set is used to train the target model, while the limited set constrains the model parameters to increase the model’s ability to associate the low-cost modality with the ground truth. In domain adaptation, there is a target dataset which consists of a few samples and a source dataset consisting of plenty of samples. If the target data is too small to build a reliable model in training, source data can be augmented to make the model more robust. Else, the target model could be trained with few examples, while a second source model is used to help make the target model more generalizable

Transformer models have brought a new and largely successful approach to alignment, sparking widespread interest in their applications in biomedical use. Transformers for NLP have also engendered new interest in Large Language Models (LLMs), which are already being applied to biomedical contexts (Tinn et al., 2023) and probing new questions about its potential use as a knowledge base for biomedical questions (Sung et al., 2021).

### Co-learning

In this last section exploring recent research in multimodal machine learning, the area of co-learning is examined, a field which has recently garnered a strong momentum in both unimodal and multimodal domains. In multimodal co-learning, knowledge learned from one modality is often used to assist learning of a second modality. This first modality which transfers knowledge is often leveraged only at train-time but is not required at test-time. Co-learning is classified in Baltrusaitis et al. (2019) as either parallel or non-parallel. In *parallel* co-learning, paired samples of modalities which share the same instance are fed into a co-learning model. By contrast, in *non-parallel* co-learning, both modalities are included in a model but are not required to be paired.

While co-learning can embody a variety of topics such as conceptual grounding and zero-shot learning, this work focuses on the use of transfer learning in biomedicine. In *multimodal transfer learning*, a model trained on a higher quality or more plentiful modality is employed to assist in the training of a model designed for a second modality which is often noisier or smaller in sample size. Transfer learning can be conducted in both parallel and non-parallel paradigms. This work focuses on one parallel form of transfer learning called privileged learning, and one non-parallel form of transfer learning called domain adaptation. A visual representation of these approaches be seen in Fig. 4.

#### Privileged Learning

*Privileged learning* originates from the mathematician Vladmir Vapnik and his ideas of knowledge transfer with the support vector machine for privileged learning (SVM+) model (Vapnik and Vashist, 2009). The concept of privileged learning introduces the idea that predictions for a low-signal, low-cost modality can be assisted by incorporating a high-signal, high-cost modality (privileged information) in training only, while at test-time only the low-cost modality is needed. Vapnik and Vashist (2009), Vapnik illustrates this concept through the analogy of a teacher (privileged information) distilling knowledge to a student (low-cost modality) before the student takes a test. Although a useful concept, the field is relatively under-explored compared to other areas of co-learning. One challenge to applying privileged learning models was that Vapnik’s SVM+ model was one of few available before the widespread use of neural networks. Furthermore, it demands that the modality deemed “privileged” must confer high accuracy on its own in order to ensure that its contribution to the model is positive. Since then, neural networks have encouraged newer renditions of privileged information models that allow more flexibility of use (Lambert et al., 2018; Shaikh et al., 2020; Sabeti et al., 2021).

Recently, privileged learning has emerged as a growing subset of biomedical literature, and understandably so. Many multimodal models today require health care professionals to gather a slew of patient information and are not trained to handle missing data. Therefore, the ability to minimize the number of required input data while still utilizing the predictive power of multiple modalities can be useful in real-world clinical settings. Hu et al. (2020) for example, the authors attempt to train a segmentation network where at train-time the “teacher network” contains four MR image modalities, but at test-time the “student network” contains only T1-weighted images, the standard modality used in preoperative neurosurgery and radiology. Chauhan et al. (2020), chest x-rays and written text from their respective radiology reports are used to train a model where only chest x-rays are available at test-time.

In privileged models based on traditional approaches (before deep neural networks), privileged information can be embedded in the model either through an alteration of allowable error (“slack variables” from SVM+) (Vapnik and Vashist, 2009), or through decision trees constructed with non-privileged features to mimic the discriminative ability of privileged features (Random Forest+) (Warner et al., 2022; Moradi et al., 2016). In a deep learning model, privileged learning is often achieved through the use of additional loss functions which attempt to constrain latent and output vectors from the non-privileged modality to mimic those from the combined privileged and non-privileged models (Hu et al., 2020; Xing et al., 2022). For example, in Chauhan et al. (2020), encoders for each modality are compared and cross entropy loss is calculated for each modality separately. The sum of these allows the chest x-ray network to freely train for only the chest x-ray modality while being constrained through the overall loss function to borrow encoding methods from the text network, which also strives to build an accurate model.

While privileged learning models can be applied where data is missing, users should heed caution when applying models in situations where there is systematic bias in reporting. Those who train privileged models without considering subject matter may inadvertently be choosing to include all their complete data in training and their incomplete data in testing. However, in clinical scenarios, data are often incomplete because a patient either did not qualify for a test (perhaps their condition was seen as not “dire enough” to warrant a test) or their situation was too serious to require a test (for example, a patient in septic shock may not pause to undergo a chest x-ray because they are in the middle of a medical emergency). Therefore, while applying data to highly complex models is a common approach in computer science, the context of the data and potential underlying biases need to be considered first to ensure a practical and well-developed model.

#### Domain Adaptation

*Domain adaptation* has been shown to be useful in biomedical data science applications where a provided dataset may be too small or costly to utilize for more advanced methods such as deep learning, but where a somewhat similar (albeit larger) dataset can be trained by such methods. The smaller dataset for which we want to train the model is called the “target” dataset and the larger dataset which will be used to assist the model with the learning task and provide better contextualization is called the “source” dataset. Domain adaptation strategies are often tailored to single modalities such as camera imaging or MRI, where measurements of an observed variable differ based on an instrument’s post-processing techniques or acquisition parameters (Xiong et al., 2020; Varsavsky et al., 2020; Yang et al., 2020). However, the distinct characteristics arising from disparate instruments or acquisition settings can lead to considerable shifts in data distribution and feature representations, mirroring the challenges faced in true multimodal contexts. Therefore, the discussion of uni-modal domain adaptation is a relevant starting point for multimodal domain adaptation, as it covers approaches to mitigate significant deviations within data that may seem similar but are represented differently. Additionally, understanding how to mitigate the impact of such variations helps one to understand ways to construct multimodal machine learning systems that confront similar challenges. We also discuss relevant multimodal domain adaptation approaches in biomedicine, which have typically consisted of applying CT images as a source domain to train an MRI target model or vice versa (Chiou et al., 2020; Xue et al., 2020; Pei et al., 2023; Jafari et al., 2022; Dong et al., 2022).

One way to train a model to adapt to different domains is through augmentation of the input data, which “generalizes” the model to interpret outside of the domain of the original data. Xiong et al. (2020), a data augmentation framework for fundus images in diabetic retinopathy (DR) is proposed to offset the domain differences of utilizing different cameras. The authors show that subtracting local average color, blurring, adaptive local contrast enhancement, and a specialized principal component analysis (PCA) strategy can increase both $R^2$ values for age prediction and DR classification area under the receiver operating curve (AUROC) on test sets where either some domain information is known *a priori* and also where no information is known, respectively. In another method which attempts to augment the source domain into more examples in the target style, Chiou et al. (2020) split the source image into latent content and style vectors, using the content vectors in a style-transfer model reminiscent of cycleGAN to feed as examples with the target domain into a segmentation network (Zhu et al., 2017). In other applications, data augmentation for domain generalization may be executed utilizing simpler affine transformations (Varsavsky et al., 2020). This demonstrates the utility of data augmentation strategies in more broadly defining decision boundaries where target domains differ from the source.

A second strategy for domain adaptation involves constraining neural network functions trained on a target domain by creating loss functions which require alignment with a source domain model. Varsavsky et al. (2020), a framework for adapting segmentation models at test-time is proposed, whereby an adversarial loss trains a target-based U-Net to be as similar to a source-based U-Net as possible. Then a paired-consistency loss with adversarial examples is utilized to fine-tune the decision boundary to include morphologically similar data points. In a specificially multimodal segmentation-based model, Xue et al. (2020) attempts to create two side-by-side networks, a segmenter and an edge generator, which both encourage the source and target output to be as similar as possible to each other. In the final loss function, the edge generator is used to constrain the segmenter in such a way as to promote better edge consistency in the target domain. In yet another, simpler example, domain adaptation to a target domain is performed in Hu et al. (2021) by taking a network trained on the source domain and simply adjusting the parameters of the batch normalization layer.

Domain adaptation in biomedicine can be a common problem where instrument models or parameters change. Among multimodal co-learning methods, most networks are constructed as segmentation networks for MRI and CT because they are similar imaging domains, although measuring different things. While CT carries distinct meaning in its pixels (measured in Hounsfield Units), MRI pixel intensities are not standardized and usually require normalization, which could pose challenges to this multimodal problem. Additionally, MRI carries much more detail than CT scans, which necessitates the model to understand contextual boundaries of objects much more than a unimodal case with only CT or MRI.

## Discussion

The rapidly evolving landscape of artificial intelligence (AI) both within the biomedical field and beyond has posed a substantial challenge in composing this survey. Our aim is to provide the reader with a comprehensive overview of the challenges and contemporary approaches to multimodal machine learning in image-based, clinically relevant biomedicine. However, it is essential to acknowledge that our endeavor cannot be fully comprehensive due to the dynamic nature of the field and the sheer volume of emerging literature within the biomedical domain and its periphery. This robust growth has led to a race among industry and research institutions to integrate the latest cutting-edge models into the healthcare sector, with a particular emphasis on the introduction of “large language models” (LLMs). In recent years, there has been an emergence of market-level insights into the future of healthcare and machine learning, as exemplified by the incorporation of machine learning models into wearable devices such as the Apple Watch and Fitbit devices for the detection of atrial fibrillation (Perino et al., 2021; Lubitz et al., 2022). This begs the question: *where does this transformative journey lead us?*

Healthcare professionals and physicians already embrace the concept of multimodal cognitive models in their diagnostic and prognostic practices, signaling that such computer models based on multimodal frameworks are likely to endure within the biomedical landscape. However, for these models to be effectively integrated into clinical settings, they must exhibit flexibility that aligns with the clinical environment. If the ultimate goal is to seamlessly incorporate these AI advancements into clinical practice, a fundamental question arises: how can these models be practically implemented on-site? Presently, most available software tools for clinicians are intended as auxiliary aids, but healthcare professionals have voiced concerns regarding the potential for increased computational workload, alert fatigue, and the limitations imposed by Electronic Health Record (EHR) interfaces (Ruiter et al., 2015; Ancker et al., 2017). Therefore, it is paramount to ensure that any additional software introduced into clinical settings serves as an asset rather than a hindrance.

Another pertinent issue emerging from these discussions pertains to the dynamics between clinical decision support systems (CDSS) and healthcare providers. What occurs when a computer-generated recommendation contradicts a physician’s judgment? This dilemma is not new, as evidenced by a classic case recounted by Evans et al. (1998), where physicians were granted the choice to either follow or disregard a CDSS for antibiotic prescription. Intriguingly, the group provided with the choice exhibited suboptimal performance compared to both the physician-only and computer-only groups. Consequently, it is unsurprising that some healthcare professionals maintain a cautious approach to computer decision support systems (Adamson and Welch, 2019; Silcox et al., 2020). Questions arise regarding the accountability of physicians if they ignore a correct computer-generated decision and the responsibility of software developers if a physician follows an erroneous computer-generated recommendation.

A pivotal ingredient notably under-represented in many CDSS models, which could help alleviate discrepancies between computer-generated and human decisions, is the incorporation of uncertainty quantification, grounded calibration, interpretability and explainability. These factors have been discussed in previous literature, underscoring the critical role of explainability in ensuring the long-term success of CDSS-related endeavors (Reddy, 2022; Khosravi et al., 2022; Kwon et al., 2020; Abdar et al., 2021).

The domain of multimodal machine learning for medically oriented image-based clinical support has garnered increasing attention in recent years. This interest has been stimulated by advances in computer science architecture and computing hardware, the availability of vast and publicly accessible data, innovative model architectures tailored for limited datasets, and the growing demand for applications in clinical and biomedical contexts. Recent studies have showcased the ability to generate synthetic images in one modality based on another (as outlined in Sect. 2.3), align multiple modalities (Sect. 2.4), and transfer latent features from one modality to train another (Sect. 2.5), among other advancements. These developments offer a promising outlook for a field that is still relatively new. However, it is also imperative to remain vigilant regarding the prevention of data biases and under-representation in ML models to maximize the potential of these technologies.

Despite these promising developments, the field faces significant hurdles, notably the lack of readily available “big data” in the medical domain. For instance, the routine digitization of histopathology slides remains a challenging goal in many healthcare facilities. Data sharing among medical institutions is fraught with challenges around appropriate procedures for the responsible sharing of patient data under institutional, national and international patient privacy regulations.

Advancing the field will likely entail overcoming these hurdles, ensuring more extensive sharing of de-identified data from research publications and greater participation in establishment of standardized public repositories for data. Dissemination of both code and pretrained model weights would also enable greater knowledge-sharing and repeatability. Models that incorporate uncertainty quantification, explainability, and strategies to account for missing data are particularly advantageous. For more guidance on building appropriate multimodal AI models in healthcare, one can refer to the World Health Organization’s new ethics and governance guidelines for large multimodal models (World Health Organization, 2024).

In conclusion, the field of multimodal machine learning in biomedicine has experienced rapid growth in each of its challenge areas of representation, fusion, translation, alignment, and co-learning. Given the recent advancements in deep learning models, escalating interest in multimodality, and the necessity for multimodal applications in healthcare, it is likely that the field will continue to mature and broaden its clinical applications. In this ever-evolving intersection of AI and healthcare, the imperative for responsible innovation resonates strongly. The future of multimodal machine learning in the biomedical sphere presents immense potential but also mandates a dedication to ethical principles encompassing data privacy, accountability, and transparent collaboration between human professionals and AI systems. As we navigate this transformative journey, the collective effort, ethical stewardship, and adherence to best practices will ensure the realization of the benefits of AI and multimodal machine learning, making healthcare more efficient, accurate, and accessible, all while safeguarding the well-being of patients and upholding the procedural and ethical standards of clinical practice.

## Data Availability

No data outside of those referenced has been used in this survey. Key papers have been summarized in Table 1.
